# Widespread Climate Change in the Himalayas and Associated Changes in Local Ecosystems

**DOI:** 10.1371/journal.pone.0036741

**Published:** 2012-05-15

**Authors:** Uttam Babu Shrestha, Shiva Gautam, Kamaljit S. Bawa

**Affiliations:** 1 University of Massachusetts, Boston, Massachusetts, United States of America; 2 Harvard University Herbarium, Harvard University, Cambridge, Massachusetts, United States of America; 3 Department of Biostatistics, Beth Israel Deaconess Medical Center, Harvard Medical School, Boston, Massachusetts, United States of America; 4 Sustainability Science Program, Harvard University, Cambridge, Massachusetts, United States of America; 5 Ashoka Trust for Research in Ecology and Environment (ATREE), Bangalore, India; Ohio State University, United States of America

## Abstract

**Background:**

Climate change in the Himalayas, a biodiversity hotspot, home of many sacred landscapes, and the source of eight largest rivers of Asia, is likely to impact the well-being of ∼20% of humanity. However, despite the extraordinary environmental, cultural, and socio-economic importance of the Himalayas, and despite their rapidly increasing ecological degradation, not much is known about actual changes in the two most critical climatic variables: temperature and rainfall. Nor do we know how changes in these parameters might impact the ecosystems including vegetation phenology.

**Methodology/Principal Findings:**

By analyzing temperature and rainfall data, and NDVI (Normalized Difference Vegetation Index) values from remotely sensed imagery, we report significant changes in temperature, rainfall, and vegetation phenology across the Himalayas between 1982 and 2006. The average annual mean temperature during the 25 year period has increased by 1.5°C with an average increase of 0.06°C yr^−1^. The average annual precipitation has increased by 163 mm or 6.52 mmyr^−1^. Since changes in temperature and precipitation are immediately manifested as changes in phenology of local ecosystems, we examined phenological changes in all major ecoregions. The average start of the growing season (SOS) seems to have advanced by 4.7 days or 0.19 days yr^−1^ and the length of growing season (LOS) appears to have advanced by 4.7 days or 0.19 days yr^−1^, but there has been no change in the end of the growing season (EOS). There is considerable spatial and seasonal variation in changes in climate and phenological parameters.

**Conclusions/Significance:**

This is the first time that large scale climatic and phenological changes at the landscape level have been documented for the Himalayas. The rate of warming in the Himalayas is greater than the global average, confirming that the Himalayas are among the regions most vulnerable to climate change.

## Introduction

The Himalayas, which represents the major parts of the Greater Hindu-Kush Himalayan mountain system, extends in an arc about 3000 kilometers in length and covers ∼750,000 km^2^ of northern Pakistan, Nepal, Bhutan, and the northwestern and northeastern states of India (**Fig. S1**) [1]. Climatic, topographic, geological, and altitudinal variations have generated unique landscapes, ecosystems, and biota in the Himalayas. Of the 825 ecoregions in the world, 13 are represented in the Himalayas [2]. This immense biological diversity is matched by cultural and ethnic diversity. Himalayas is also the source of the 8 largest rivers of Asia and is known as “water tower of Asia” [3]; the rivers and their tributaries sustain about 1.4 billion people [4]. Thus climate change in the region is a matter of global concern.

Much of the recent discussion about climate change in the Himalayas has been dominated by the extent of glacial melting [5], [6]; however, glaciers have not been systematically monitored [7]. The IPCC report predicts large scale changes in temperature and precipitation over the Asian land mass [8]. Limited studies on temperature or precipitation for a few localized places show that warming in the Himalayas is 3 times greater than the global average [3]. However, changes at the regional level remain to be documented. Furthermore, the impacts of climate change on phenological patterns are not well understood due to lack of historical ground-based observations on phenology. Thus despite the fact that the Himalayas are among the regions most vulnerable to climate change [3], have unique biodiversity, and are undergoing rapid environmental change [9], there is no systematic analysis of climate change and its effects on ecosystems and biodiversity, nor on hydrology, agriculture, and livelihoods in this important and extraordinary region of the world.

We used global mean monthly surface air temperature [10], precipitation [11], and GIMMS-NDVI dataset [12] from the year 1982 to 2006 for 13 ecoregions of the Himalayas. The global mean monthly surface air temperature dataset of 0.5-degree grid is produced from ground-based weather station data collected from the Global Historical Climatology Network version 2 and the Climate Anomaly Monitoring System using interpolation methods. The quality of this dataset is found to be reasonably good in comparison with several other ground-based land surface temperature datasets, and it captures most of the common temporal-spatial features observed in climatology both at regional and global scale [10]. Similarly, we used precipitation data produced by Climate Prediction Center Merged Analysis of Precipitation (CMAP), the 2.5-degree gridded global monthly precipitation data were produced by the combination of different sources: gauge observations and estimates inferred from a variety of satellite observations [11]. The GIMMS-NDVI dataset is corrected for different sources of non-vegetation error introduced by inter-sensor calibration, orbital drift, cloud cover, solar angle differences, volcanic eruptions, and other atmospheric contaminations [12]–[13]. Long term temporal and large spatial coverage of this data make it possible to detect trends in vegetation phenology [14]–[16]. The extracted data were analyzed in a GIS framework to detect spatio-temporal changes in climate. Since changes in temperature and precipitation are directly manifested as changes in phenology of local ecosystems, we examined the impact of such changes on the onset of growing season and senescence in the 13 different ecoregions of the Himalayas. Furthermore, we correlated changes in phenological parameters with changes in climatic variables.

IPCC predicts that average annual mean temperature over the Asian land mass, including the Himalayas, will increase by about 3°C by the 2050 s and about 5°C by the 2080 s [8], [17]. Similarly, average annual precipitation in this region will increase by 10–30% by 2080 [8]. During the last few decades, the Himalayas have experienced increasing temperature [3], [18], [19]. However, data on precipitation are not consistent; the precipitation has increased in some areas but decreased in other areas [3], [20]. Changes in temperature and precipitation have influenced phenology of plants [21]–[23]. Although basic research on ecological responses, including phenology, to climatic change is substantially lacking in the Himalayas, it is generally anticipated that climate change in this region may alter phenology at both individual species and community levels [3]. Our results demonstrate significant changes in temperature and precipitation of the Himalayas–greater than the upper bounds predicted by IPCC and the recent Indian assessments [24]. Furthermore, we demonstrate that landscape-level changes in the phenology of local ecosystems all across the Himalayas are well correlated with changes in climate.

## Methods

NDVI dataset of Global Inventory Modeling and Mapping Studies (GIMMS) is publicly available for a 25 year period spanning from 1982 to 2006. To make a consistent comparison of temperature, precipitation and phenological parameters, we used global mean monthly temperature [10], precipitation [11], and GIMMS-NDVI [12] dataset from 1982 to 2006 of the Himalayas. The temperature, precipitation and NDVI datasets have resolution of 0.5°, 2.5° and 64 km^2^ respectively. The temperature and precipitation data were interpolated into the spatial resolution of NDVI data (64 km^2^) using bilinear interpolation method, which involves resampling a raster cell using the weighted average of the four nearest cells to determine a new cell value.

GIMMS-NDVI datasets are derived from the Advanced Very High Resolution Radiometer (AVHRR) and corrected to remove non vegetation effects such as aerosol, cloud, volcanic and sensor degradation [12]. However, we discard the areas where average annual NDVI values are less than 0.1 as non-vegetated regions to minimize the further errors and consider pixels with an average annual NDVI values greater than 0.1 for further analysis [13], [25], [26].

In the phenology literature, about a dozen of different methods have been proposed for calculating the start and end of the growing season using time series of the satellite images [27]. Yet, there is a lack of universally accepted definition of spring onset [27], [28]. These methods are grouped into four different categories: thresholds, derivatives, smoothing functions and fitted models [27]. In this study, we determined NDVI threshold values for SOS and EOS from NDVI climatology (seasonal NDVI cycle) by computing the rates of maximum ascend and descend of NDVI and used that NDVI threshold values to determine yearly SOS and EOS [23], [26], [29]. This method may reduce non climate effects such as aerosols, clouds, disturbances, and defoliation on annual values [29].

**Figure 1:**
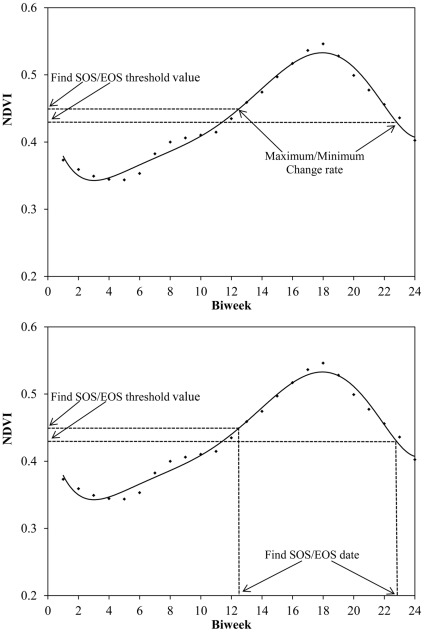
Climatology of a single pixel and schematic of retrieving of SOS and EOS (Modified from [**29**]).

The 25-year mean annual NDVI climatological cycle of a single pixel and schematic of the method is given in the **Fig. 1**. We first calculated 25-year average annual NDVI climatological cycle for each pixel then determined the rate of changes in the NDVI climatological cycle by calculating NDVI_ratio_. We calculated NDVI_ratio_ using the formula, NDVI_ratio_ for n^th^ time period  =  (NDVI (n+1) – NDVI (n))/NDVI (n). We detected the time (n) with maximum NDVI_ratio_ and corresponding NDVI (n) as the NDVI threshold for the SOS (start of the season) date. Similarly, we determined the time (n) with minimum NDVI_ratio_ and corresponding NDVI (n+1) at time (n+1) as the NDVI threshold for the EOS (end of the season) date.

We used these NDVI threshold values (SOS and EOS) derived from 25-year average annual NDVI climatology data to calculate the SOS and EOS in each year. We calculated the dates for each year on which the NDVI thresholds of SOS and EOS are passed by estimating the daily NDVI value by fitting the 15 days NDVI seasonal curve with the 6^th^ degree polynomial equation:

NDVI = α+α_1_x^1^+α_2_x^2^+α_3_x^3^+….α_n_x^n^, where x is the Julian days of the year.

This polynomial equation also helps to smooth the abnormal value. The R^2^ value in about 97% pixels is more than 0.90. We used a least square regression analysis to establish the relationship between the 15 days NDVI time-series data from January to July and from July to December and the corresponding Julian day to represent the seasonal changes in NDVI as a function of Julian day.

LOS is determined by defining the period between SOS and EOS. With the yearly Julian days of phenological events (SOS and EOS), we analyzed their trends over the 25 years from 1982 to 2006 using a linear regression.

We also investigated the relationship between timing of phenological events (SOS and EOS) and climatic variables (temperature and precipitation) by calculating correlation coefficients using Pearson’s correlation. We measured the correlation between climatic variables (average temperature and cumulative precipitation) of the pre-growing-season period (between December to April) and the annual SOS. Similarly, we measured the correlation between climatic variables (average temperature and cumulative precipitation) of the growing-season period (between mean SOS and EOS date, i.e. June to October) and the annual EOS.

We analyzed temperature and precipitation in the spatial domain of the ecoregions and of the whole study area to observe the annual and seasonal trends over 25 years. In case of phenological parameters, we derived trends at individual pixel level, ecoregion level, and whole study area. Spatial average of ecoregions and the whole study area were carried out after we derived yearly SOS and EOS of individual pixels.

## Results

**Figure 2:**
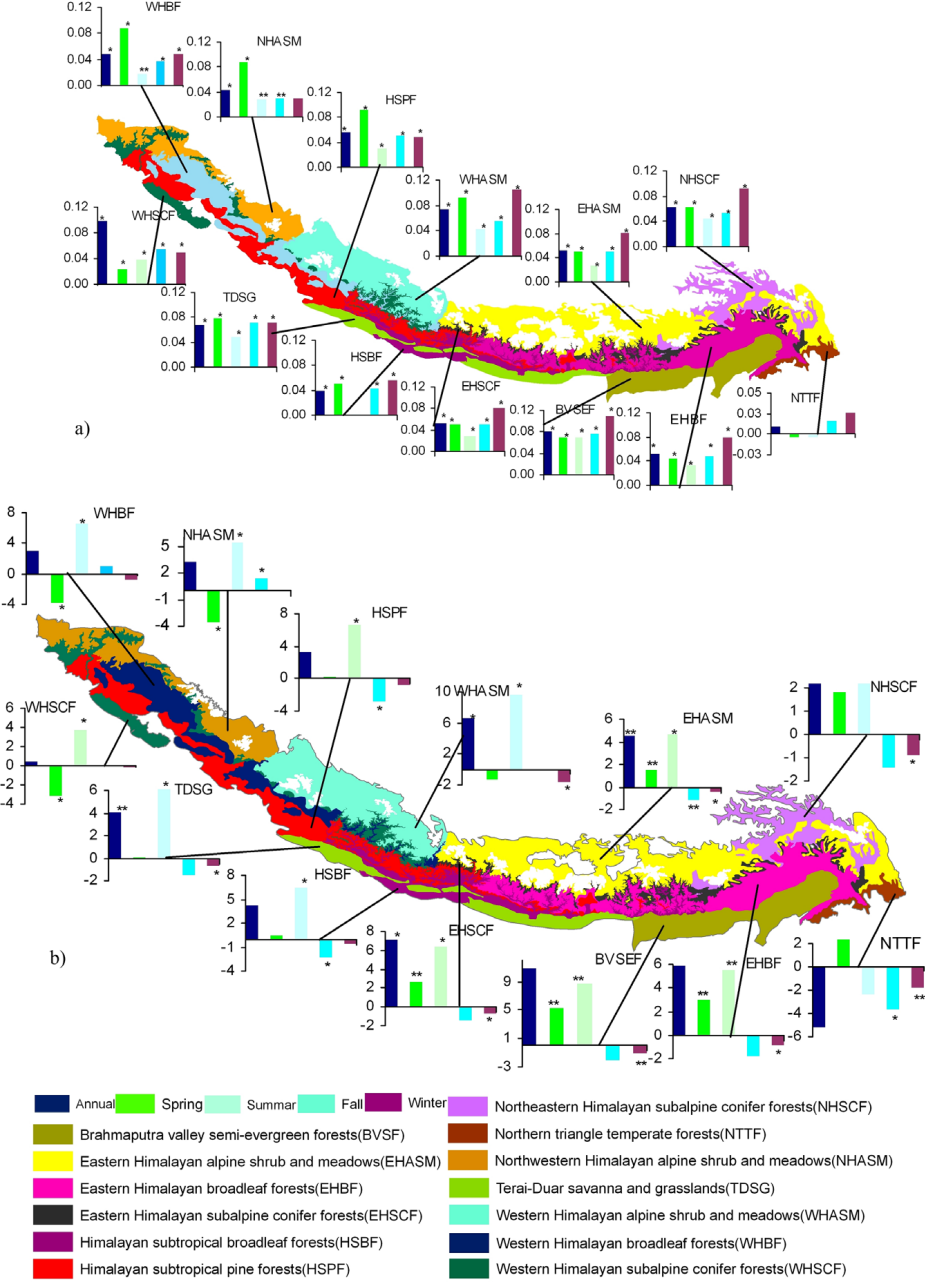
Temperature and Precipitation changes in the Himalayas. **a.** Annual and season trends of temperature (°C yr^−1^, shown in bar) in different ecoregions **b.** Annual and season trends of precipitation (mm yr^−1^, shown in bar) in different ecoregions (*P≤0.05, **P≤0.10).

Our results indicate the Himalayas have warmed by 1.5°C from 1982 to 2006, at an average rate of 0.06°C yr^−1^ (R^2^ = 0.66, p = <0.01), but the rate of warming varies across seasons and ecoregions. The greatest increase, 1.75°C, is observed in winter with an average increase of 0.07°C yr^−1^ (R^2^ = 0.43, p = <0.01), whereas the least increase, 0.75°C, is in summer, average increase is 0.03°C yr^−1^ (R^2^ = 0.45, p = <0.01). The Brahmaputra Valley semi-evergreen forest ecoregion has experienced the greatest rate of warming, 2.0°C (0.08°C yr^−1^), and the Northern Triangle temperate forest ecoregion, the least, 0.25°C (0.01°C yr^−1^) (**Fig. 2a**).

Average annual precipitation has increased by 163 mm (6.52 mm yr^−1^) (R^2^ = 0.21, p = 0.02) during the 25-year period. This increase has largely resulted from an increase of 187 mm (7.48 mm yr^−1^) (R^2^ = 0.39, p = <0.01) during the summer months (June, July and August), balanced by a decrease of 17 mm (−0.68 mm yr^−1^) (R^2^ = 0.08, p = 0.16) in winter (December, January, and February). The pattern of increase in annual precipitation is consistent in all ecoregions except the Northern Triangle temperate forest ecoregion. The Brahmaputra Valley semi-evergreen forest ecoregion had the greatest increase, 269.25 mm (10.77 mm yr^−1^), whereas the Northern Triangle temperate forest had the greatest decrease, 130.5 mm (−5.22 mm yr^−1^) (**Fig. 2b**).

**Figure 3:**
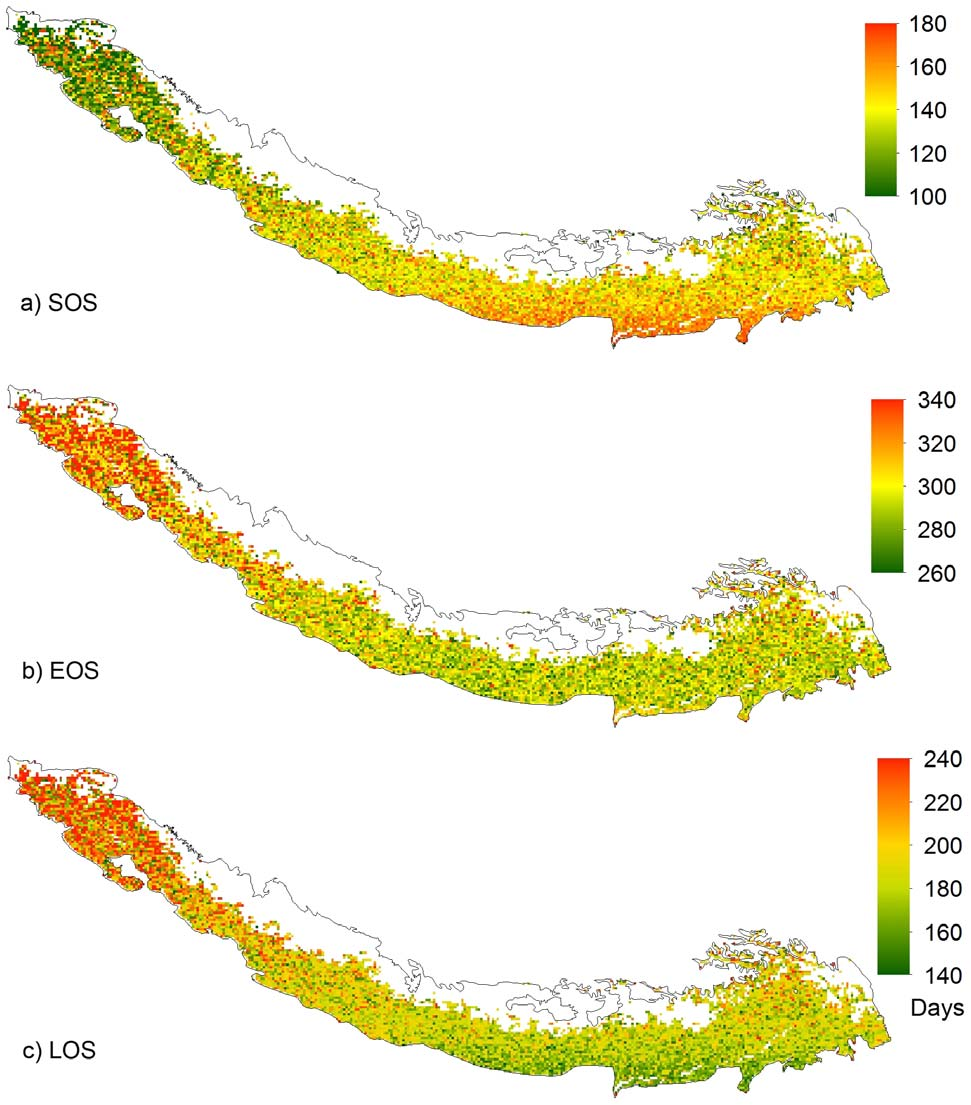
Spatial distribution of 25 year average SOS, EOS and LOS in the Himalayas.

Spatial distribution of the 25 year average phenological events (SOS, EOS, and LOS) or climatology for the Himalayas from 1982 to 2006 is given in **Fig. 3**. The average SOS dates in **Fig. 3a** ranges from 100 days (about April 10) to 180 days (about June 29) in different regions. Western Himalayas shows earlier SOS and then greenness extends towards the southeastern part of the Himalayas later in the year. Similarly, the average EOS dates ranges from 260 days (about September 17) to 340 days (about December 6) are shown in **Fig. 3b**. Late EOS is seen in the western parts; however, mixed patterns are observed in the central and eastern region. The early SOS and late EOS make the length of LOS in western parts of the Himalayas longer. The overall LOS ranges from 140 to 240 days in the study area (**Fig. 3c**).

**Figure 4:**
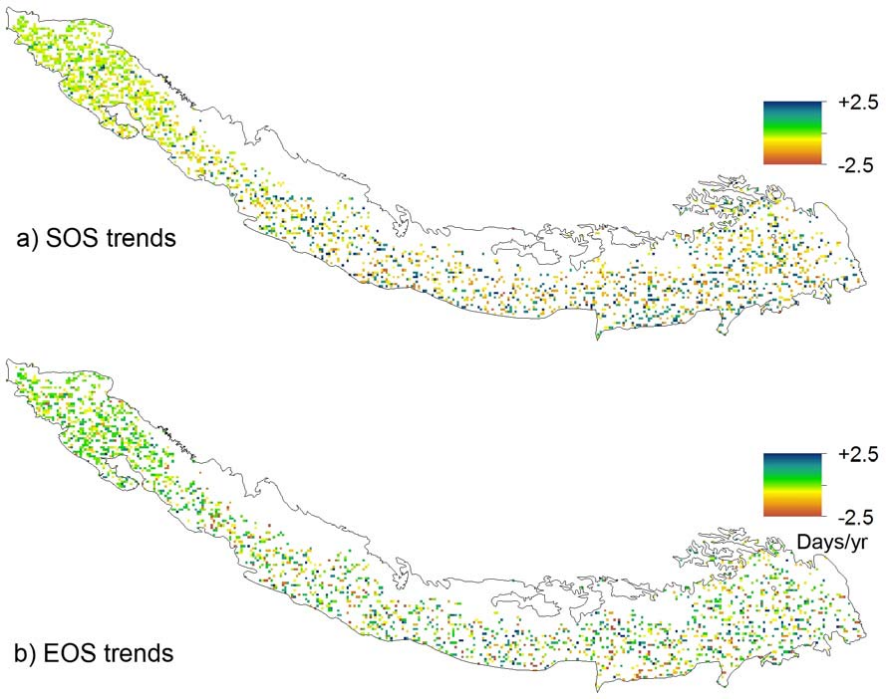
Spatial distribution of significant (P<0.10) SOS and EOS trends in the Himalayas from 1982 –**2006.**

**Figure 5:**
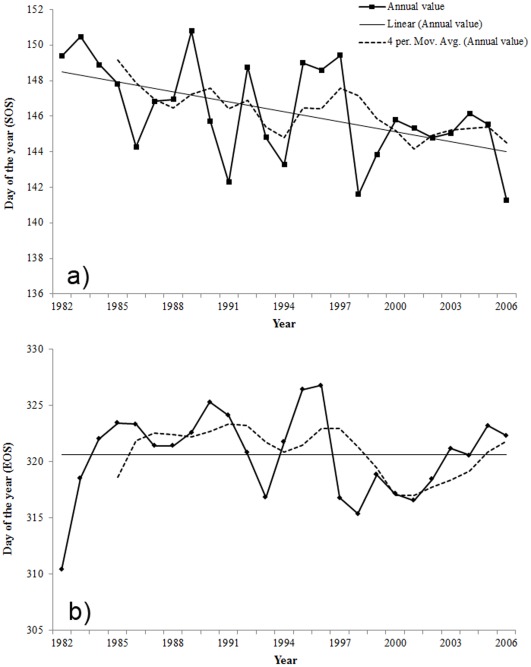
Annual variations and trends of spatially-averaged SOS and EOS in the Himalayas from 1982 –**2006.**

Spatial distribution of significant trends in phenological parameters (SOS and EOS) for the Himalayas over 25 years from 1982–2006 are given in **Fig. 4**. To evaluate the annual trends in the phenological parameters, those parameters were analyzed in the spatial domain of the whole study area and of the ecoregions. Phenology has changed significantly during the 25-year period; the average SOS has significantly advanced by 4.7 days (−0.19 days yr^−1^, R^2^ = 0.26, P = <0.01) over the whole study area (**Fig. 5a**). Most of the ecoregions (8 out of 13) in the study area show a significant advancement in SOS (**Table 1**). The greatest advancement (0.47days days yr^−1^, R^2^ = 0.64, P = <0.01) is observed in Northwestern Himalayan alpine shrub and meadows.

**Table 1:** Linear trends of area-averaged phenological parameters (SOS, EOS and LOS) in different ecoregions of the Himalayas.

Ecoregions	SOS Trends(days yr^−1^)	EOS Trends(days yr^−1^)	LOS Trends (days yr^−1^)
Brahmaputra Valley semi-evergreen forests	−0.01	−0.03	−0.02
Eastern Himalayan alpine shrub and meadows	−0.26*	0.07	0.34
Eastern Himalayan broadleaf forests	−0.21*	0.07	0.28
Eastern Himalayan subalpine conifer forests	−0.22*	0.11	0.33
Himalayan subtropical broadleaf forests	−0.14	0.03	0.17
Himalayan subtropical pine forests	−0.23*	−0.03	0.20
Northeastern Himalayan subalpine conifer forests	0.00	−0.03	−0.03
Northern Triangle temperate forests	−0.02	0.03	0.05
Northwestern Himalayan alpine shrub and meadows	−0.47*	−0.20	0.27
Terai-Duar savanna and grasslands	−0.04	0.05	0.10
Western Himalayan alpine shrub and Meadows	0.01	−0.02	−0.03
Western Himalayan broadleaf forests	−0.31*	−0.08	0.23
Western Himalayan subalpine conifer forests	−0.31*	−0.01	0.30

* Significant ≤0.05.

The linear trend of spatial-average EOS shows that there is no change in EOS in the whole study area over the 25-year period (**Fig. 5b**). Both positive and negative trends in EOS are observed at ecoregion levels. However, none of these are significant (**Table 1**). The positive linear trend of LOS in the whole study area shows the lengthening of the growing season by 4.7 days in 25 years. Although both increases and decreases in LOS are observed at the ecoregion levels, the increasing trend is more pervasive (**Table 1**). Advancement in SOS and no change of EOS have caused overall increases in LOS and the largest increase in LOS is observed in the Eastern Himalayan alpine shrub and meadows (**Table 1**). Spatially, significant trends are observed in the ecoregions of the higher elevation whereas ecoregions in the lower elevation such as Brahmaputra Valley semi-evergreen forests, Himalayan subtropical broadleaf forest, Northern Triangle temperate forests, Terai-Duar savanna and grasslands do not show significant changes in SOS. Shorter LOS in the lowermost parts of the central and eastern region is perhaps due to higher proportion of agricultural fields in those areas.

**Figure 6:**
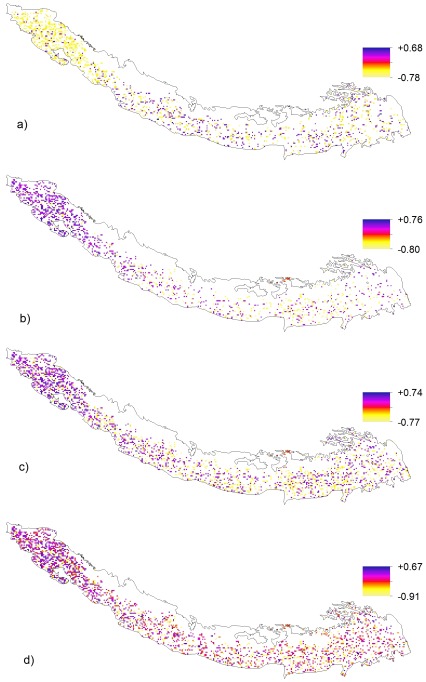
Spatial distribution of significant (P≤0.10) correlation coefficient of SOS and EOS related temperature and precipitation. **a**) correlation coefficient between SOS with average temperature of March and April **b**) correlation coefficient between SOS with cumulative precipitation of March and April **c**) correlation coefficient between EOS with average temperature of August, September and October **d**) correlation coefficient between EOS with precipitation of October.

To observe the relationship between phenological cycle and climate, the average temperature and cumulative precipitation were correlated with SOS and EOS dates. We calculated correlations between SOS dates and the average temperature and cumulative precipitation of lag period of 1–5 month (December to April) before the SOS. We found maximum significant correlations of SOS with average temperature and cumulative precipitation of 2 months prior to average SOS dates (**Fig. 6a**). Ecosystem response to the climatic variables vary among the pixels however, negative correlation between average temperature of pre-season and SOS days is widespread, about 55% pixels show negative correlations (of which about 35% are significantly negative) vs. about 43% pixels show positive correlations (of which about 14% are significantly positive). Spatially, the negative correlation is pronounced in the western parts of the Himalaya. Regarding the rainfall, we found widespread correlations in the western Himalayas highlighting the sensitivity of this relatively dry region to the precipitation (**Fig. 6b**).

Similarly, we examined the correlation between average EOS and temperature and precipitation. We also calculated correlations between EOS dates and the average temperature and cumulative precipitation of lag period of 1–5 month ( June to October) before the average EOS date. Maximum correlations were observed between average EOS date (**Fig. 6c** **)** and the average temperature of three months prior to EOS and cumulative precipitation of one month prior to EOS (**Fig. 6d**). Both the positive and negative correlations were observed; however, the percentage of negative correlation (68%) between EOS and precipitation of one month prior to EOS date was common, with 26% pixels having significant correlation.

## Discussion

Global mean surface temperature has increased on average by 0.8°C in last century and by 0.6°C in past three decades from 1975 to 2005 [30]. Our results show that the temperature increase in the Himalayas from 1982–2006 is 1.5°C (0.06°C yr^−1^), considerably higher than the global average for the comparable, but longer period (1975–2005). Our results for temperature are similar to those inferred for the Greater Hindu-Kush Himalayan region including the Tibetan plateau [3]. Previous work in the Himalayas has been highly localized; small-scale studies based on meteorological stations data in the Western [31], Central [18] and Eastern Himalayas [32], [33] show consistent warming trends. Warming trends reported previously in the Himalayas are: increases of 0.04°C yr^−1^ during 1962–2004 in Kullu Valley in the Western Himalayas [31], 0.06°C yr^−1^ during 1977–2000 in Nepal in the Central Himalayas [18], and 0.03°C yr^−1^ from 1985 to 2002 in Bhutan in the Eastern Himalayas [32], [33].

For precipitation, both increasing and decreasing trends in Eastern Himalayas [34], lack of a consistent trend in Central Himalayas [20], and decreasing trends in Western Himalayas [35] have been reported. However, we found a consistent trend of increasing average precipitation in the Himalayas as a whole, and also in most of the ecoregions except the Northern Triangle temperate forest ecoregion. Similarly, IPCC 2007 describes increasing trends of precipitation in northern and central Asia. It further predicts that average annual precipitation will increase by 10–30% on the Tibetan Plateau as a whole by 2080 [8]. Likewise, a net 60–206 mm (5–13%) increase in precipitation over the simulated 1970 s rainfall in the Indian Himalayas by 2030 s is projected [24].

Regarding phenology, our results showing the advancement of the start of the growing season (SOS) are consistent with similar studies in the Alps (1.53 days yr^−1^) [14] and in the mountains of East Asia [15]. There is evidence based on satellite derived NDVI data that the onset of spring vegetation greening has advanced by 0.79 days yr^−1^ in China and there is strong linear relationship between spring temperature and start of the growing season [25]. We also found the maximum negative correlation between SOS and average temperature of the preceding two months in our study area. European ground-based phenological observations based on more than 125,000 records show similar advances (0.25 days yr^−1^) in onset of growing period and such advancements are correlated with increases in winter and spring temperatures, presumably linked with climate change [21]. A 1°C warming in mean temperature of early spring could produce a 7.5-day early onset of the growing season [22], [25].

Although, significant advancement of SOS was observed at the regional and ecoregion level, at the pixel level, both advancement and delay of SOS were observed. However, the frequency of the significant negative trends (advancement) of SOS is much greater than that of positive trends (delay). A clear spatial pattern can be observed, higher proportion of significant trends of SOS is observed in the ecoregions of the higher elevation (Northwestern Himalayan alpine shrub and meadows, Western Himalayan subalpine conifer forests, Eastern Himalayan alpine shrub and meadows) than in the ecoregions of lower elevation. Similarly, the average rate of increase of temperature is higher in the ecoregions of the higher elevation. Sharma et al. (2009) also report greater warming at higher elevations in the Himalayas [36]. Furthermore, a larger proportion of local people at higher elevations seem to be experiencing climate change than people at lower elevations [37]. At the pixel level, the negative correlation is pronounced in the western parts of the Himalaya–the region, with greater changes in average temperature. Other studies also show that higher mean temperature in late winter or early spring may cause the early onset of growing period [22]. The relationship between temperature and SOS, however, might be location dependent.

Landscape level phenological observations are lacking in the Himalayas, but field-based observations on rhododendrons in the Himalayas show that some species have started to flower a month earlier than in the past [3]. Phenophases of eleven tree species in the Western Himalayas (Himachal Pradesh)–including leaf emergence, flower initiation and growing period–have shifted over eight years though the authors do not specify the time frame of observations [24].

Our results of prolonged LOS whole study area and ecoregion level are consistent with the findings of the other places such as in Europe by 0.8 days yr^−1^ [15], in China by 1.16 days yr^−1^ [25]. However, in America, an increase of LOS was found due to advancement of EOS but with no change in SOS [38] Localized study in central China using NDVI data showed lengthening of growing seasons [39]. Extension in LOS might be caused by enhanced net primary production induced by increase in temperature [25], [40]. However, both increases and decreases of LOS were found at pixel level. This type of nuances have been noted in the previous studies [14], [29] based on satellite derived NDVI data. Given the diverse and extensive nature of Himalayan vegetation and forests due to large altitudinal gradients, our results are not unusual. Further ground based observations for specific regions are required to confirm the results.

Our results provide some insights into the relationship between precipitation and phenology. We observed higher proportion of positive correlation of SOS and cumulative precipitation of two months (March-April) prior to SOS in the western Himalayas. The western Himalayas gets more precipitation in spring than the eastern part. Similarly, the higher proportion of negative correlations between EOS and precipitation of a month prior to average EOS indicates a link between the late monsoon precipitation and EOS. End of the season might be advanced by increased precipitation late in the growing season. Late precipitation may enhance growth, reducing nutrient availability for continuing vegetative growth [41].

Overall, we have documented landscape-scale climatic and phenological changes in the Himalayas. We should however keep in mind that the satellite-derived vegetation phenology provides spatially and species-aggregated coarse-grained information, and may not correspond exactly to ground-based phenological observations. Nevertheless, it is useful for regions for which ground-based data are meager or lacking. Biases due to satellite drift, incomplete corrections, aerosol contamination and human interruption are a common in satellite derived NDVI datasets [29]. However, comparison of satellite-derived phenology with ground-based observation on the Tibetan plateau shows that the data can be reliably used to derive onset of growing season and senescence [23]. It is interesting that although ground-based measurements are lacking, there is widespread belief among local communities that the type of climate and phenological changes we have observed are common and are impacting their agriculture and lives [37], [42]. Clearly, future research with ground validation, cross-calibration, and longer time series with better spatial and temporal resolution are necessary to understand changes in climate and associated phenological patterns, and the consequences of such changes on biodiversity, agriculture and people. Overall, our results indicate that the Himalayas are indeed among the regions most vulnerable to climate change.

## Supporting Information

Figure S1 **Map of the Himalayas and Greater Hindu Kush Himalayan Region.**
(TIF)
