# Supplementary figures and images for: Widespread Climate Change in the Himalayas and Associated Changes in Local Ecosystems
Source: PLoS One. 2012 May 15;7(5):e36741. doi: 10.1371/journal.pone.0036741 (PMC3352921; doi:10.1371/journal.pone.0036741)

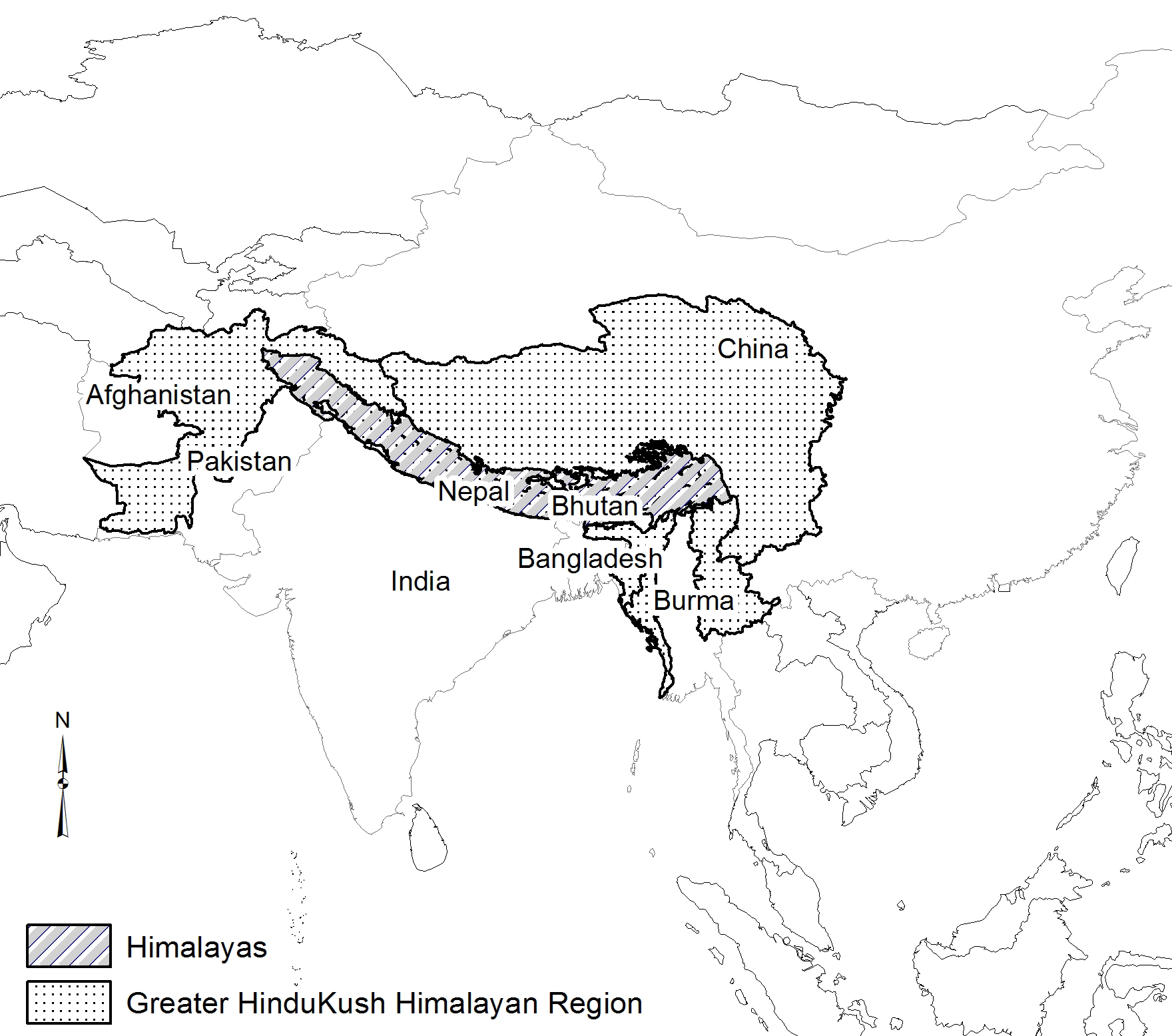

Supplement: Figure S1 — Map of the Himalayas and Greater Hindu Kush Himalayan Region. (TIF) [file pone.0036741.s001.tif]
